# Viscount Knutsford on Nurses' Hours

**Published:** 1920-03-06

**Authors:** 

**Affiliations:** Kneesworth Hall, Royston, Herts.


### THE EDITOR'S LETTER-BOX.

**(Correspondence on all subjects is invited, but we cannot in any way be responsible for the opinions expressed by our correspondents, who must give their name and address as a guarantee of good faith, but not necessarily for publication. Correspondents are reminded that brevity of style and conciseness of statement greatly facilitate early insertion.]**

### Viscount Knutsford on Nurses' Hours.

*To the Editor of* The Hospital.

Sir,—"Ierne" seems to have an extraordinary facility for misunderstanding me. Perhaps it is my fault. Stating my views as "Ierne" does would lead your readers to believe that I had advocated a twelve-hours' day of work for nurses. Of course, I never said or wrote anything of the sort. I did write that in private nursing the twenty-four hours could be well managed by two nurses—and so it can, as everybody knows. If "Ierne" had ever had an operation (I have had four) he would know this. But in saying this I did not mean that each nurse was to be on duty the whole twelve hours, as "Ierne" would have your readers believe. Out of those twelve hours would be deducted two or three hours off and meals. "Ierne" might just as well write that, because I wrote that for a confinement one nurse was able to care for mother and baby that a twenty-four hours' day was right for nurses. It is a pity to mislead.—Yours faithfully,

Knutsford.

Kneesworth Hall, Royston, Herts.

